# 2,4,5-Trichloro­anilinium perchlorate 18-crown-6 clathrate

**DOI:** 10.1107/S1600536812006162

**Published:** 2012-02-17

**Authors:** Jie Xu

**Affiliations:** a Ordered Matter Science Research Center, College of Chemistry and Chemical Engineering, Southeast University, Nanjing 210096, People’s Republic of China

## Abstract

In the title compound, C_6_H_5_Cl_3_N^+^·ClO_4_^−^·C_12_H_24_O_6_, the perchlorate anion is disordered over two orientations in a 0.666 (17):0.334 (17) ratio. The ammonium group of the organic cation inserts into the crown ether ring and forms three bifurcated N—H⋯(O,O) hydrogen bonds to generate a supra­molecular complex. The macrocycle has approximate *D*_3*d*_ local symmetry.

## Related literature

For background to mol­ecular ferroelectric materials, see: Fu *et al.* (2011 ▶).
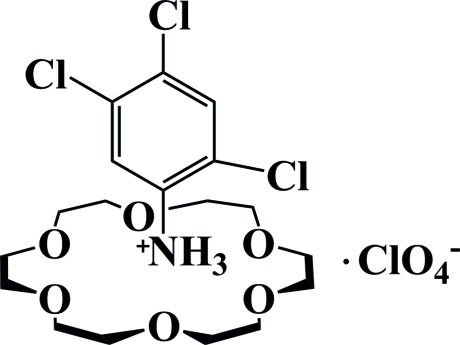


## Experimental

### 

#### Crystal data

C_6_H_5_Cl_3_N^+^·ClO_4_^−^·C_12_H_24_O_6_
*M* *_r_* = 561.22
Triclinic, 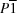

*a* = 9.4961 (19) Å
*b* = 11.783 (2) Å
*c* = 11.852 (2) Å
α = 97.86 (3)°
β = 90.39 (3)°
γ = 105.26 (3)°
*V* = 1266.1 (4) Å^3^
*Z* = 2
Mo *K*α radiation
μ = 0.52 mm^−1^
*T* = 298 K
0.10 × 0.05 × 0.05 mm

#### Data collection

Rigaku Mercury2 (2 × 2 bin mode) diffractometer
Absorption correction: multi-scan (*CrystalClear*; Rigaku, 2005 ▶) *T*_min_ = 0.910, *T*_max_ = 1.000
11310 measured reflections
4713 independent reflections
3562 reflections with *I* > 2σ(*I*)
*R*_int_ = 0.042

#### Refinement

*R*[*F* ^2^ > 2σ(*F* ^2^)] = 0.054
*wR*(*F* ^2^) = 0.152
*S* = 1.03
4713 reflections
346 parameters
18 restraints
H-atom parameters constrained
Δρ_max_ = 0.57 e Å^−3^
Δρ_min_ = −0.28 e Å^−3^

### 

Data collection: *CrystalClear* (Rigaku, 2005 ▶); cell refinement: *CrystalClear*; data reduction: *CrystalClear*; program(s) used to solve structure: *SHELXS97* (Sheldrick, 2008 ▶); program(s) used to refine structure: *SHELXL97* (Sheldrick, 2008 ▶); molecular graphics: *SHELXTL* (Sheldrick, 2008 ▶); software used to prepare material for publication: *SHELXTL*.

## Supplementary Material

Crystal structure: contains datablock(s) I, global. DOI: 10.1107/S1600536812006162/hb6625sup1.cif

Structure factors: contains datablock(s) I. DOI: 10.1107/S1600536812006162/hb6625Isup2.hkl

Supplementary material file. DOI: 10.1107/S1600536812006162/hb6625Isup3.cml

Additional supplementary materials:  crystallographic information; 3D view; checkCIF report

## Figures and Tables

**Table 1:** Hydrogen-bond geometry (Å, °)

*D*—H⋯*A*	*D*—H	H⋯*A*	*D*⋯*A*	*D*—H⋯*A*
N1—H1*C*⋯O1^i^	0.89	2.13	2.916 (3)	147
N1—H1*C*⋯O6^i^	0.89	2.21	2.899 (3)	134
N1—H1*D*⋯O5^i^	0.89	2.06	2.896 (3)	156
N1—H1*D*⋯O4^i^	0.89	2.52	3.052 (3)	119
N1—H1*E*⋯O3^i^	0.89	2.19	3.046 (3)	161
N1—H1*E*⋯O2^i^	0.89	2.35	2.856 (3)	116

Symmetry code: (i) 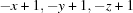.

**Fig. 1.**
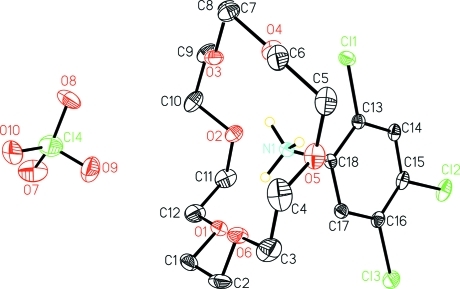
A view of the title compound with displacement ellipsoids drawn at the 50% probability level.

**Fig. 2.**
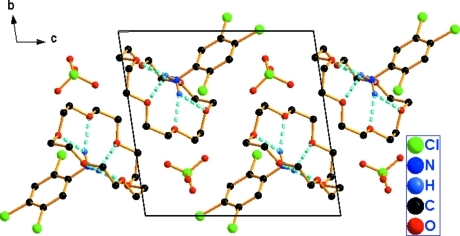
The crystal packing of the title compound, showing the H-bonding interactions. H atoms not involved in hydrogen bonding (dashed line) have been omitted for clarity.

## supplementary crystallographic information

### Comment

With the purpose of obtaining phase transition crystals of amino compounds, various amines have been studied and we have elaborated a series of new materials with this organic molecules (Fu *et al.* 2011). In this study, we describe the crystal structure of the title compound, (I).

The title compound was composed of cationic [(C_6_H_5_NCl_3_).(C_12_H_24_O_6_)]^+^ and one ClO_4_^-^ anion (Fig.1). Supramolecular cation was assembled by protonated 2,4,5-trichloroanilinium and 18-crown-6 through six strong N—H···O hydrogen bonds. The C-N bonds of cation were almost perpendicular to the mean oxygen planes of crown ethers. The macrocycle adopts a conformation with approximate D_3d_ symmetry, with all O-C-C-O torsion angles being *gauche* and alternating in sign, and all C-O-C-C torsion angles being *anti*. Supramolecular cation structure, [(C_6_H_5_NCl_3_).(C_12_H_24_O_6_)]^+^, was introduced as counter cation to ClO_4_^-^ anion. Cl has a flattened tetrahedral coordination by four O atoms. The ClO_4_^-^ anion is disordered over two sets of sites with refined occupancies 0.666 (17) and 0.334 (17).

The title compound was stabilized by intermolecular N—H···O hydrogen bonds, the ClO_4_^-^ anion not participating in the H-bonding interactions. The intermolecular N—H···O H-bonding length are within the usual range of 2.856 (3) to 3.052 (3)Å. (Table 1 and Fig.2).

### Experimental

18-Crown-6 (6 mmol), HClO_4_ (6 mmol) and 2,4,5-trichloroaniline (3 mmol) were dissolved in water/EtOH (1:1 *v*/*v*) solvent. The solution was slowly evaporated in air affording colourless blocks.

### Refinement

All H atoms attached to C atoms were fixed geometrically and treated as riding with C–H = 0.93 Å (C-aromatic) and 0.98 Å (C-methylene), with *U_iso_*(H)=1.2*U_eq_*(C). The positional parameters of the H atoms (N1) were intially refined freely, subsequently restrained using a distance of N–H = 0.89 (2) Å, and in the final refinements treated in riding motion of their parent nitrogen atom with *U_iso_*(H)=1.5*U_eq_*(N).

The ClO_4_^-^ anion is disordered over sites and refined using the PART instruction in SHELXL (Sheldrick, 2008)

### Crystal data

**Table:**

C_6_H_5_Cl_3_N^+^·ClO_4_^−^·C_12_H_24_O_6_	*Z* = 2
*M**_r_* = 561.22	*F*(000) = 584
Triclinic, *P*1	*D*_x_ = 1.472 Mg m^−^^3^
Hall symbol: -P 1	Mo *K*α radiation, λ = 0.71073 Å
*a* = 9.4961 (19) Å	Cell parameters from 4713 reflections
*b* = 11.783 (2) Å	θ = 3.1–26.5°
*c* = 11.852 (2) Å	µ = 0.52 mm^−^^1^
α = 97.86 (3)°	*T* = 298 K
β = 90.39 (3)°	Block, colourless
γ = 105.26 (3)°	0.10 × 0.05 × 0.05 mm
*V* = 1266.1 (4) Å^3^	

### Data collection

**Table:**

Rigaku Mercury2 (2x2 bin mode) diffractometer	4713 independent reflections
Radiation source: fine-focus sealed tube	3562 reflections with *I* > 2σ(*I*)
Graphite monochromator	*R*_int_ = 0.042
Detector resolution: 13.6612 pixels mm^-1^	θ_max_ = 25.5°, θ_min_ = 3.1°
CCD profile fitting scans	*h* = −11→11
Absorption correction: multi-scan (*CrystalClear*; Rigaku, 2005)	*k* = −14→14
*T*_min_ = 0.910, *T*_max_ = 1.000	*l* = −14→14
11310 measured reflections	

### Refinement

**Table:**

Refinement on *F*^2^	Secondary atom site location: difference Fourier map
Least-squares matrix: full	Hydrogen site location: inferred from neighbouring sites
*R*[*F*^2^ > 2σ(*F*^2^)] = 0.054	H-atom parameters constrained
*wR*(*F*^2^) = 0.152	*w* = 1/[σ^2^(*F*_o_^2^) + (0.070*P*)^2^ + 0.575*P*] where *P* = (*F*_o_^2^ + 2*F*_c_^2^)/3
*S* = 1.03	(Δ/σ)_max_ < 0.001
4713 reflections	Δρ_max_ = 0.57 e Å^−^^3^
346 parameters	Δρ_min_ = −0.28 e Å^−^^3^
18 restraints	Extinction correction: *SHELXL97* (Sheldrick, 2008), Fc^*^=kFc[1+0.001xFc^2^λ^3^/sin(2θ)]^-1/4^
Primary atom site location: structure-invariant direct methods	Extinction coefficient: 0.019 (2)

### Special details

**Table:**

Geometry. All esds (except the esd in the dihedral angle between two l.s. planes) are estimated using the full covariance matrix. The cell esds are taken into account individually in the estimation of esds in distances, angles and torsion angles; correlations between esds in cell parameters are only used when they are defined by crystal symmetry. An approximate (isotropic) treatment of cell esds is used for estimating esds involving l.s. planes.
Refinement. Refinement of F^2^ against ALL reflections. The weighted R-factor wR and goodness of fit S are based on F^2^, conventional R-factors R are based on F, with F set to zero for negative F^2^. The threshold expression of F^2^ > 2sigma(F^2^) is used only for calculating R-factors(gt) etc. and is not relevant to the choice of reflections for refinement. R-factors based on F^2^ are statistically about twice as large as those based on F, and R- factors based on ALL data will be even larger.

### Fractional atomic coordinates and isotropic or equivalent isotropic displacement parameters (Å^2^)

**Table:**

	*x*	*y*	*z*	*U*_iso_*/*U*_eq_	Occ. (<1)
Cl1	0.30933 (8)	0.67394 (7)	0.38541 (7)	0.0584 (3)	
N1	0.5852 (2)	0.7317 (2)	0.25875 (17)	0.0421 (5)	
H1C	0.6761	0.7384	0.2370	0.063*	
H1D	0.5358	0.7570	0.2078	0.063*	
H1E	0.5417	0.6559	0.2637	0.063*	
Cl2	0.60060 (12)	1.00366 (8)	0.72283 (6)	0.0763 (3)	
C18	0.5882 (3)	0.8037 (2)	0.3703 (2)	0.0366 (6)	
Cl3	0.86985 (10)	1.06897 (8)	0.56512 (8)	0.0800 (3)	
C13	0.4666 (3)	0.7804 (2)	0.4375 (2)	0.0408 (6)	
C15	0.5973 (4)	0.9285 (3)	0.5859 (2)	0.0492 (7)	
C16	0.7156 (3)	0.9555 (3)	0.5175 (2)	0.0489 (7)	
O3	0.5578 (2)	0.53489 (19)	0.78658 (19)	0.0616 (6)	
O1	0.1138 (2)	0.2827 (2)	0.7236 (2)	0.0636 (6)	
C17	0.7111 (3)	0.8924 (2)	0.4094 (2)	0.0458 (6)	
H17A	0.7908	0.9100	0.3635	0.055*	
C14	0.4716 (3)	0.8432 (2)	0.5456 (2)	0.0467 (7)	
H14A	0.3908	0.8279	0.5906	0.056*	
O2	0.3401 (3)	0.43429 (19)	0.61331 (18)	0.0643 (6)	
O4	0.6757 (3)	0.3898 (2)	0.90636 (19)	0.0747 (7)	
O6	0.2005 (3)	0.1902 (2)	0.9069 (2)	0.0697 (6)	
O5	0.4919 (3)	0.1617 (2)	0.93064 (19)	0.0773 (7)	
C1	0.0241 (4)	0.2633 (4)	0.8172 (3)	0.0762 (11)	
H1A	−0.0777	0.2451	0.7919	0.091*	
H1B	0.0459	0.3346	0.8730	0.091*	
C9	0.5364 (5)	0.5924 (3)	0.6920 (3)	0.0813 (12)	
H9A	0.5927	0.5699	0.6289	0.098*	
H9B	0.5691	0.6780	0.7131	0.098*	
C10	0.3801 (5)	0.5564 (3)	0.6579 (3)	0.0776 (11)	
H10A	0.3230	0.5705	0.7234	0.093*	
H10B	0.3611	0.6027	0.6007	0.093*	
C6	0.6873 (5)	0.3373 (4)	1.0053 (3)	0.0837 (12)	
H6A	0.6265	0.3631	1.0634	0.100*	
H6B	0.7877	0.3616	1.0352	0.100*	
C8	0.7075 (4)	0.5679 (3)	0.8253 (4)	0.0799 (11)	
H8A	0.7437	0.6538	0.8398	0.096*	
H8B	0.7656	0.5379	0.7674	0.096*	
C7	0.7188 (4)	0.5178 (4)	0.9294 (4)	0.0877 (13)	
H7A	0.8187	0.5450	0.9605	0.105*	
H7B	0.6565	0.5448	0.9856	0.105*	
C2	0.0499 (4)	0.1641 (4)	0.8693 (3)	0.0841 (13)	
H2A	−0.0118	0.1507	0.9337	0.101*	
H2B	0.0249	0.0922	0.8142	0.101*	
C3	0.2348 (6)	0.1026 (4)	0.9654 (4)	0.0952 (14)	
H3A	0.2244	0.0297	0.9131	0.114*	
H3B	0.1678	0.0855	1.0261	0.114*	
C11	0.1938 (5)	0.3922 (4)	0.5702 (4)	0.0843 (12)	
H11A	0.1828	0.3183	0.5193	0.101*	
H11B	0.1721	0.4495	0.5261	0.101*	
C12	0.0878 (4)	0.3719 (4)	0.6604 (4)	0.0899 (13)	
H12A	0.0972	0.4455	0.7115	0.108*	
H12B	−0.0107	0.3460	0.6265	0.108*	
C5	0.6391 (5)	0.2059 (4)	0.9751 (3)	0.0909 (13)	
H5A	0.7031	0.1814	0.9190	0.109*	
H5B	0.6493	0.1700	1.0426	0.109*	
C4	0.3898 (7)	0.1477 (5)	1.0147 (4)	0.1070 (17)	
H4A	0.4008	0.2236	1.0625	0.128*	
H4B	0.4087	0.0923	1.0625	0.128*	
Cl4	0.0719 (14)	0.7626 (15)	0.7497 (13)	0.128 (4)	0.334 (17)
O10	0.040 (2)	0.7880 (19)	0.8570 (11)	0.121 (7)	0.334 (17)
O9	0.053 (3)	0.653 (2)	0.703 (3)	0.152 (7)	0.334 (17)
O8	0.2286 (19)	0.797 (2)	0.7517 (18)	0.116 (6)	0.334 (17)
O7	−0.056 (2)	0.790 (2)	0.6899 (19)	0.143 (7)	0.334 (17)
Cl4'	0.0736 (4)	0.7704 (3)	0.7438 (2)	0.0580 (11)	0.666 (17)
O10'	0.0532 (11)	0.7151 (19)	0.8447 (12)	0.190 (7)	0.666 (17)
O8'	−0.0154 (17)	0.8368 (11)	0.7385 (16)	0.194 (6)	0.666 (17)
O7'	0.0428 (16)	0.6824 (13)	0.6488 (9)	0.170 (5)	0.666 (17)
O9'	0.2038 (16)	0.8551 (14)	0.7353 (12)	0.192 (7)	0.666 (17)

### Atomic displacement parameters (Å^2^)

**Table:**

	*U*^11^	*U*^22^	*U*^33^	*U*^12^	*U*^13^	*U*^23^
Cl1	0.0438 (4)	0.0646 (5)	0.0609 (5)	0.0076 (3)	0.0091 (3)	0.0018 (4)
N1	0.0388 (11)	0.0521 (13)	0.0324 (11)	0.0092 (10)	0.0006 (9)	0.0014 (9)
Cl2	0.1349 (9)	0.0625 (5)	0.0344 (4)	0.0375 (5)	0.0029 (4)	−0.0051 (3)
C18	0.0411 (14)	0.0425 (14)	0.0288 (12)	0.0160 (11)	0.0007 (10)	0.0040 (10)
Cl3	0.0662 (6)	0.0702 (6)	0.0860 (7)	0.0096 (4)	−0.0143 (5)	−0.0312 (5)
C13	0.0474 (15)	0.0392 (14)	0.0388 (14)	0.0152 (12)	0.0034 (11)	0.0084 (11)
C15	0.080 (2)	0.0444 (16)	0.0293 (13)	0.0286 (15)	−0.0014 (13)	0.0032 (11)
C16	0.0540 (17)	0.0456 (16)	0.0457 (16)	0.0170 (13)	−0.0099 (13)	−0.0055 (12)
O3	0.0480 (12)	0.0607 (13)	0.0674 (14)	0.0004 (10)	0.0010 (10)	0.0068 (11)
O1	0.0443 (12)	0.0651 (14)	0.0767 (15)	0.0104 (10)	0.0073 (11)	0.0020 (11)
C17	0.0441 (15)	0.0490 (16)	0.0422 (15)	0.0127 (12)	0.0014 (12)	−0.0010 (12)
C14	0.0670 (18)	0.0451 (16)	0.0352 (14)	0.0247 (14)	0.0150 (13)	0.0108 (12)
O2	0.0764 (15)	0.0543 (13)	0.0599 (13)	0.0113 (11)	−0.0039 (11)	0.0126 (10)
O4	0.0741 (16)	0.0865 (18)	0.0550 (14)	0.0184 (13)	−0.0222 (12)	−0.0125 (12)
O6	0.0624 (14)	0.0651 (15)	0.0746 (15)	0.0004 (11)	0.0200 (12)	0.0182 (12)
O5	0.103 (2)	0.0890 (18)	0.0466 (13)	0.0389 (15)	−0.0023 (13)	0.0064 (12)
C1	0.0423 (18)	0.092 (3)	0.079 (2)	0.0084 (18)	0.0038 (17)	−0.022 (2)
C9	0.113 (3)	0.052 (2)	0.065 (2)	−0.005 (2)	0.015 (2)	0.0152 (17)
C10	0.121 (3)	0.051 (2)	0.060 (2)	0.021 (2)	−0.015 (2)	0.0118 (16)
C6	0.088 (3)	0.106 (3)	0.056 (2)	0.042 (2)	−0.0287 (19)	−0.019 (2)
C8	0.056 (2)	0.065 (2)	0.097 (3)	−0.0067 (17)	−0.0035 (19)	−0.020 (2)
C7	0.071 (2)	0.089 (3)	0.081 (3)	0.006 (2)	−0.030 (2)	−0.033 (2)
C2	0.064 (2)	0.094 (3)	0.065 (2)	−0.024 (2)	0.0227 (18)	−0.004 (2)
C3	0.130 (4)	0.076 (3)	0.076 (3)	0.011 (3)	0.031 (3)	0.031 (2)
C11	0.088 (3)	0.081 (3)	0.086 (3)	0.018 (2)	−0.025 (2)	0.027 (2)
C12	0.058 (2)	0.076 (3)	0.139 (4)	0.023 (2)	−0.024 (2)	0.020 (3)
C5	0.111 (3)	0.103 (3)	0.067 (2)	0.054 (3)	−0.031 (2)	−0.008 (2)
C4	0.151 (5)	0.135 (4)	0.061 (3)	0.072 (4)	0.016 (3)	0.035 (3)
Cl4	0.090 (6)	0.144 (8)	0.143 (8)	0.028 (6)	−0.025 (6)	0.004 (7)
O10	0.135 (11)	0.177 (15)	0.031 (6)	0.018 (10)	0.037 (6)	−0.010 (8)
O9	0.148 (11)	0.102 (10)	0.203 (17)	0.057 (9)	−0.002 (13)	−0.030 (12)
O8	0.058 (6)	0.188 (15)	0.089 (7)	0.017 (9)	0.016 (5)	0.010 (10)
O7	0.117 (9)	0.173 (15)	0.138 (11)	0.020 (10)	−0.047 (9)	0.060 (10)
Cl4'	0.0618 (17)	0.0635 (16)	0.0419 (15)	0.0072 (12)	0.0088 (11)	0.0024 (10)
O10'	0.089 (5)	0.337 (18)	0.144 (9)	−0.002 (8)	−0.019 (5)	0.153 (11)
O8'	0.194 (12)	0.141 (8)	0.264 (15)	0.093 (8)	−0.029 (10)	−0.008 (8)
O7'	0.235 (10)	0.144 (9)	0.114 (6)	0.056 (7)	−0.074 (6)	−0.049 (6)
O9'	0.149 (10)	0.187 (11)	0.152 (9)	−0.087 (8)	0.067 (8)	−0.022 (8)

### Geometric parameters (Å, º)

**Table:**

Cl1—C13	1.724 (3)	C10—H10A	0.9700
N1—C18	1.466 (3)	C10—H10B	0.9700
N1—H1C	0.8900	C6—C5	1.486 (6)
N1—H1D	0.8900	C6—H6A	0.9700
N1—H1E	0.8900	C6—H6B	0.9700
Cl2—C15	1.735 (3)	C8—C7	1.454 (6)
C18—C17	1.374 (4)	C8—H8A	0.9700
C18—C13	1.395 (4)	C8—H8B	0.9700
Cl3—C16	1.733 (3)	C7—H7A	0.9700
C13—C14	1.383 (4)	C7—H7B	0.9700
C15—C14	1.376 (4)	C2—H2A	0.9700
C15—C16	1.383 (4)	C2—H2B	0.9700
C16—C17	1.385 (4)	C3—C4	1.511 (7)
O3—C8	1.425 (4)	C3—H3A	0.9700
O3—C9	1.427 (4)	C3—H3B	0.9700
O1—C1	1.409 (4)	C11—C12	1.473 (6)
O1—C12	1.441 (4)	C11—H11A	0.9700
C17—H17A	0.9300	C11—H11B	0.9700
C14—H14A	0.9300	C12—H12A	0.9700
O2—C11	1.412 (4)	C12—H12B	0.9700
O2—C10	1.413 (4)	C5—H5A	0.9700
O4—C6	1.415 (5)	C5—H5B	0.9700
O4—C7	1.442 (5)	C4—H4A	0.9700
O6—C3	1.422 (5)	C4—H4B	0.9700
O6—C2	1.436 (4)	Cl4—O9	1.30 (2)
O5—C4	1.391 (5)	Cl4—O10	1.322 (19)
O5—C5	1.426 (5)	Cl4—O8	1.43 (2)
C1—C2	1.468 (6)	Cl4—O7	1.53 (2)
C1—H1A	0.9700	Cl4'—O8'	1.300 (13)
C1—H1B	0.9700	Cl4'—O9'	1.384 (11)
C9—C10	1.470 (6)	Cl4'—O7'	1.395 (10)
C9—H9A	0.9700	Cl4'—O10'	1.428 (12)
C9—H9B	0.9700		
			
C18—N1—H1C	109.5	O3—C8—H8B	110.0
C18—N1—H1D	109.5	C7—C8—H8B	110.0
H1C—N1—H1D	109.5	H8A—C8—H8B	108.4
C18—N1—H1E	109.5	O4—C7—C8	110.2 (3)
H1C—N1—H1E	109.5	O4—C7—H7A	109.6
H1D—N1—H1E	109.5	C8—C7—H7A	109.6
C17—C18—C13	120.2 (2)	O4—C7—H7B	109.6
C17—C18—N1	120.1 (2)	C8—C7—H7B	109.6
C13—C18—N1	119.7 (2)	H7A—C7—H7B	108.1
C14—C13—C18	120.1 (3)	O6—C2—C1	110.1 (3)
C14—C13—Cl1	119.9 (2)	O6—C2—H2A	109.6
C18—C13—Cl1	120.1 (2)	C1—C2—H2A	109.6
C14—C15—C16	120.6 (2)	O6—C2—H2B	109.6
C14—C15—Cl2	118.0 (2)	C1—C2—H2B	109.6
C16—C15—Cl2	121.3 (2)	H2A—C2—H2B	108.2
C15—C16—C17	120.1 (3)	O6—C3—C4	109.4 (3)
C15—C16—Cl3	120.7 (2)	O6—C3—H3A	109.8
C17—C16—Cl3	119.2 (2)	C4—C3—H3A	109.8
C8—O3—C9	111.4 (3)	O6—C3—H3B	109.8
C1—O1—C12	114.0 (3)	C4—C3—H3B	109.8
C18—C17—C16	119.5 (3)	H3A—C3—H3B	108.2
C18—C17—H17A	120.2	O2—C11—C12	113.0 (3)
C16—C17—H17A	120.2	O2—C11—H11A	109.0
C15—C14—C13	119.3 (3)	C12—C11—H11A	109.0
C15—C14—H14A	120.3	O2—C11—H11B	109.0
C13—C14—H14A	120.3	C12—C11—H11B	109.0
C11—O2—C10	113.4 (3)	H11A—C11—H11B	107.8
C6—O4—C7	112.1 (3)	O1—C12—C11	109.9 (3)
C3—O6—C2	114.4 (3)	O1—C12—H12A	109.7
C4—O5—C5	113.4 (3)	C11—C12—H12A	109.7
O1—C1—C2	109.6 (3)	O1—C12—H12B	109.7
O1—C1—H1A	109.7	C11—C12—H12B	109.7
C2—C1—H1A	109.7	H12A—C12—H12B	108.2
O1—C1—H1B	109.7	O5—C5—C6	113.9 (3)
C2—C1—H1B	109.7	O5—C5—H5A	108.8
H1A—C1—H1B	108.2	C6—C5—H5A	108.8
O3—C9—C10	108.6 (3)	O5—C5—H5B	108.8
O3—C9—H9A	110.0	C6—C5—H5B	108.8
C10—C9—H9A	110.0	H5A—C5—H5B	107.7
O3—C9—H9B	110.0	O5—C4—C3	112.3 (4)
C10—C9—H9B	110.0	O5—C4—H4A	109.1
H9A—C9—H9B	108.3	C3—C4—H4A	109.1
O2—C10—C9	108.7 (3)	O5—C4—H4B	109.1
O2—C10—H10A	109.9	C3—C4—H4B	109.1
C9—C10—H10A	109.9	H4A—C4—H4B	107.9
O2—C10—H10B	109.9	O9—Cl4—O10	121.0 (19)
C9—C10—H10B	109.9	O9—Cl4—O8	97.3 (18)
H10A—C10—H10B	108.3	O10—Cl4—O8	104.3 (15)
O4—C6—C5	108.9 (3)	O9—Cl4—O7	98.8 (15)
O4—C6—H6A	109.9	O10—Cl4—O7	99.6 (15)
C5—C6—H6A	109.9	O8—Cl4—O7	138.5 (15)
O4—C6—H6B	109.9	O8'—Cl4'—O9'	98.7 (13)
C5—C6—H6B	109.9	O8'—Cl4'—O7'	108.0 (8)
H6A—C6—H6B	108.3	O9'—Cl4'—O7'	111.9 (9)
O3—C8—C7	108.5 (3)	O8'—Cl4'—O10'	110.5 (9)
O3—C8—H8A	110.0	O9'—Cl4'—O10'	118.0 (10)
C7—C8—H8A	110.0	O7'—Cl4'—O10'	109.0 (8)
			
C17—C18—C13—C14	−2.6 (4)	C8—O3—C9—C10	−178.9 (3)
N1—C18—C13—C14	176.2 (2)	C11—O2—C10—C9	−175.8 (3)
C17—C18—C13—Cl1	176.8 (2)	O3—C9—C10—O2	−67.2 (4)
N1—C18—C13—Cl1	−4.4 (3)	C7—O4—C6—C5	179.2 (3)
C14—C15—C16—C17	−3.1 (4)	C9—O3—C8—C7	173.2 (3)
Cl2—C15—C16—C17	178.7 (2)	C6—O4—C7—C8	179.2 (3)
C14—C15—C16—Cl3	176.1 (2)	O3—C8—C7—O4	64.0 (4)
Cl2—C15—C16—Cl3	−2.1 (3)	C3—O6—C2—C1	176.1 (3)
C13—C18—C17—C16	2.4 (4)	O1—C1—C2—O6	59.2 (4)
N1—C18—C17—C16	−176.4 (2)	C2—O6—C3—C4	−172.0 (3)
C15—C16—C17—C18	0.4 (4)	C10—O2—C11—C12	−77.7 (4)
Cl3—C16—C17—C18	−178.8 (2)	C1—O1—C12—C11	175.7 (3)
C16—C15—C14—C13	2.9 (4)	O2—C11—C12—O1	−61.4 (4)
Cl2—C15—C14—C13	−178.8 (2)	C4—O5—C5—C6	−83.1 (4)
C18—C13—C14—C15	−0.1 (4)	O4—C6—C5—O5	−59.9 (5)
Cl1—C13—C14—C15	−179.5 (2)	C5—O5—C4—C3	179.2 (3)
C12—O1—C1—C2	175.0 (3)	O6—C3—C4—O5	−65.9 (5)

### Hydrogen-bond geometry (Å, º)

**Table:**

*D*—H···*A*	*D*—H	H···*A*	*D*···*A*	*D*—H···*A*
N1—H1*C*···O1^i^	0.89	2.13	2.916 (3)	147
N1—H1*C*···O6^i^	0.89	2.21	2.899 (3)	134
N1—H1*D*···O5^i^	0.89	2.06	2.896 (3)	156
N1—H1*D*···O4^i^	0.89	2.52	3.052 (3)	119
N1—H1*E*···O3^i^	0.89	2.19	3.046 (3)	161
N1—H1*E*···O2^i^	0.89	2.35	2.856 (3)	116

Symmetry code: (i) −*x*+1, −*y*+1, −*z*+1.
